# Overexpression of the human antigen R suppresses the immediate paradoxical proliferation of melanoma cell subpopulations in response to suboptimal BRAF inhibition

**DOI:** 10.1002/cam4.1091

**Published:** 2017-06-01

**Authors:** Marylise Fernandez, Hedwig Sutterlüty‐Fall, Christoph Schwärzler, Sylvain Lemeille, Wolf‐Henning Boehncke, Rastine Merat

**Affiliations:** ^1^ Department of Pathology and Immunology University of Geneva Switzerland; ^2^ Institute of Cancer Research Medical University of Vienna Austria; ^3^ Flow Cytometry Core Facility Ecole Polytechnique Fédérale de Lausanne Switzerland; ^4^ Division of Dermatology University Hospital of Geneva Switzerland

**Keywords:** BRAF inhibitor, cell heterogeneity, melanoma, RNA‐binding protein HuR, single‐cell mass cytometry, targeted therapy

## Abstract

Tumor plasticity and the heterogeneous response of melanoma cells to targeted therapies are major limits for the long‐term efficacy of this line of therapy. Targeting tumor plasticity is theoretically possible through the modulation of the expression of RNA‐binding proteins which can affect many different compensatory mechanisms of the adaptive response of malignant cells to targeted therapies. Human antigen R (HuR) is a modulator of gene expression and a transacting factor in the mRNA‐processing machinery used in the cell stress response, and is a potential target for reducing tumor plasticity. In this experiment, we exploit the inherent heterogeneous response of the A375 melanoma line to suboptimal BRAF inhibition as a model of immediate adaptive response. We first observe that HuR overexpression can prevent the heterogeneous response and thus the immediate paradoxical proliferation induced by low‐doses vemurafenib treatment. We then use single‐cell mass cytometry to characterize subpopulations, including those that paradoxically proliferate, based on their proliferation rate and the expression patterns of markers involved in the reversible adaptive resistance to BRAF inhibition and/or recognized as HuR targets involved in cell cycle regulation. Under suboptimal BRAF inhibition, HuR overexpression affects these subpopulations and their expression pattern with contrasting responses depending on their proliferation rate: faster‐proliferating vemurafenib‐sensitive or ‐resistant subpopulations showed higher death tendency and reduced size, and slower‐proliferating subpopulations showed an attenuated resistant expression response and their paradoxical proliferation was inhibited. These observations pave the way to new therapeutic strategies for preventing the heterogeneous response of tumors to targeted therapies.

## Introduction

Impressive clinical results have shown that targeted therapies for melanoma, that is, BRAF and MEK inhibitors, can efficiently treat highly mutagenic solid malignancies by blocking critical cell survival pathways. However, subsequent clinical observations have demonstrated that these inhibitors, even when combined, are rarely curative and that resistance almost inevitably develops within a few weeks or months 1. Currently, great efforts are being deployed to understand the mechanisms behind this resistance 2, and to develop other combinatory therapies to target the reversible nongenetic adaptive resistance, documented in recent studies 3, 4, 5, 6. Therapeutic targeting and prevention of reversible nongenetic adaptive resistance is most likely possible, but any efficient strategy to prevent the emergence of compensatory mechanisms likely needs to have a simultaneous effect on multiple major nodes within the cancer signaling network 7. A sufficiently large‐scale modulation of genetic expression that can affect many different compensatory mechanisms and pathways can potentially be achieved through drug‐mediated expression modulation of RNA‐binding proteins (RBPs). RBPs play a central role in differentially controlling gene expression at the posttranscriptional level 8. These modulations can occur differentially toward mRNAs subrepertoires in malignancies as demonstrated for the translation initiation factors eIF4E and eIF3 9, 10. The RBP Human antigen R (HuR, also called HuA or ELAVL1) is estimated to have, depending on the studies, from 5000 to more than 7000 direct mRNA targets 11, 12 and within the cancer signaling network to which we refer 7, at least 10% of the nodes, including highly connected ones, belong to the HuR repertoire. The extensive modulatory effects of HuR also operate indirectly “in cascade” through the regulation of the expression of other RBPs such as the cap‐binding protein eIF4E 13. Although ubiquitously expressed 14, HuR presence within the mRNA‐processing machinery seems less obligatory than other cancer‐deregulated RBPs like, for example, eIF4E. This variability in its presence is used in the cell stress response to many environmental changes such as UV radiation 15 and thus makes HuR an excellent candidate for modulating cell plasticity and heterogeneity. Although mainly considered as a tumorigenic protein, partly because some of its targets are cell cycle‐ and apoptosis‐regulating proteins and partly because its expression pattern increases in some malignancies 16, HuR involvement in cancer is likely highly complex 17 according to the available in vivo experimental data 18, 19. HuR function, like most proteins, is regulated in part through posttranslational modifications that include phosphorylation, methylation, NEDDylation, and ubiquitination 20, but its abundance and compartmentalization are as much importantly involved in determining its stabilizing effects on most of its mRNA targets and their enhanced protein expression. In this study, we make the hypothesis that if overexpressed, HuR, through its extensive regulatory effects, can limit the compensatory mechanisms of the immediate adaptive response to BRAF inhibition. We use as an experimental approach the inherent heterogeneous response of the sensitive A375 *BRAFV600* melanoma cell line to suboptimal BRAF inhibition. Using single‐cell mass cytometry, we characterize the expression profile and behavior of various cell subpopulations within this cell line toward the BRAF inhibitor vemurafenib and under the effect of HuR overexpression, and observe that when overexpressed, HuR can overcome the emergence of paradoxically proliferative subpopulations.

## Material and Methods

### Cell lines and culture

The A375*,* Malme‐3M, and MEL‐CLS‐3 melanoma cell lines were purchased from CLS Cell Lines Service GmbH. HT‐29 colon carcinoma cell line was purchased from Sigma‐Aldrich. Cell‐line authentication for the A375 cell line was conducted by an independent laboratory (DSMZ, Leibniz‐Institute, Germany) with DNA profiling using eight different and highly polymorphic STR loci. Cells were maintained at 37°C and 5% CO_2_ in a humidified atmosphere. A375 and MEL‐CLS‐3 cells were grown in DMEM growth media supplemented with 10% FBS, 2 mmol/L glutamine, and 1% penicillin/streptomycin. Malme‐3M cells were grown in IMDM growth media supplemented with 20% FBS. HT‐29 cells were grown in RPMI growth media supplemented with 10% FBS. Cells were treated with the BRAF inhibitor vemurafenib purchased from Selleckchem and dissolved in dimethylsulphoxide (DMSO, 10 mmol/L storing concentration).

### Cell proliferation assay

Cell proliferation was measured using WST‐1 reagent (Roche applied Science). Melanoma A375 and MEL‐CLS‐3 cells were plated at 2500 cells per well, HT‐29 cells at 7500 cells per well, and Malme‐3M cells at 10,000 cells per well in 96‐well tissue culture plates (100 *μ*L medium). After 24 h, the cells were treated with vemurafenib or DMSO at the indicated concentrations in triplicate or quadruplicate. For the standard proliferation assays, after the treatment period of 72 h, WST reagent (20 *μ*L, 10% of the final volume) was added to the wells and incubated at 37°C for 1–2 h depending on the experiment in order to stay within the linear range of the assay. The plates were read at 450(−650) nm on a V max kinetic ELISA Microplate Reader (Molecular Devices). Cell proliferation is expressed as percentage of the absorbance compared with the DMSO‐treated cells. Proliferation assays for overexpression experiments were scheduled as follows: A375 cells were plated (800,000 cells per well) in six‐well culture plates. After 24 h, the cells were infected overnight at indicated m.o.i. (in 3 mL medium), transferred the next morning in 96‐well culture plates (2500 cells per well), and left for 12 h before being treated as indicated above for 72 h. For Western blot analysis, A375 cells were plated (400,000 cells per well) in six‐well culture plates and infected as described above. Protein extractions were performed 48 h postinfection. For cell cycle, cell death determination, and mass cytometry experiments, the content of each well from the six‐well plates used for overnight infection was transferred to a 10 cm culture dish and left for 24 h before being treated for either 24 h (cell cycle, cell death determination, and mass cytometry experiments) or 48 h (cell death determination) at the indicated concentrations and then harvested.

### Western blot analysis

Western blot analysis was performed on cell extracts of A375 cells at 48 h postinfection. Whole cell lysates were prepared using RIPA buffer (PBS, 1% NP40, 0.1% sodium deoxycholate, 0.1% SDS) supplemented with fresh protease and phosphatase inhibitors (easy packs tablets, Roche). Cell lysates were quantified for protein content using Bio‐Rad DC kit. Protein samples were resolved on 12.5% polyacrylamide Bis‐Tris gel (Bio‐rad) and then transferred to nitrocellulose membrane. After saturation, the membranes were incubated overnight with the anti‐HuR mouse monoclonal antibody (Santa‐Cruz/3A2‐sc‐5261, 1:1000) raised against full‐length HuR of human origin. Anti‐*α*‐tubulin mouse monoclonal antibody was used as a loading control (Sigma‐Aldrich/Clone B‐5‐1‐2, 1:10,000). The secondary antibody conjugated to horseradish peroxidase (Bio‐Rad goat antimouse IgG‐HRP, 1:3000) was incubated at room temperature for 30 min. Blots were developed using the ECL system (Thermo‐Scientific) according to the manufacturer's instructions. Cell fractionation was conducted using the NE‐PER Nuclear and Cytoplasmic Extraction Reagents (ThermoFisher Scientific) according to the manufacturer's instructions. Nuclear and cytoplasmic fractions were analyzed for HuR expression by Western blot as described above. Anti‐*α*‐tubulin and anti‐lamin A mouse monoclonal antibody (Santa‐Cruz/4A58‐sc‐7148, 1:500) were used to check the quality of the cell fractionation.

### Adenovirus construct and virus stock preparation

Adenoviruses expressing GFP or T7 epitope‐tagged (*gene 10* leader peptide) HuR were generated as previously described 21, 22. The original plasmid containing T7 epitope‐HuR was initially provided by U. Atasoy. The T7 epitope‐HuR fragment was isolated by an Nhe1/Xba1 digestion and subcloned into the Xba1 site of pAdlox vector. Successful cloning was verified by sequencing. Recombinant viruses were generated by cotransfecting pAdlox–T7 epitope‐HuR plasmid DNA, digested previously with *Sfi*I, and Ψ5 adenovirus DNA into 293‐CRE8 cells as described 21. Recombinant adenoviruses were selected by serial reinfection in 293‐CRE8 cells and finally amplified in 293 cells and purified by CsCl density‐gradient centrifugation. Infectious virus particles were calculated by determining the optical density and A375 cells were infected with an m.o.i. of 5 and 25 (an m.o.i. of 125 was also tested in Western blot analysis). AdGFP virus was generated using the same procedure after subcloning the eGFP from pEGFPC1 via Nde1/EcoR1 into pAdlox.

### Cell death quantification and cell cycle analysis

Cells were harvested using Versene, transferred to FACS tubes, and stained using the propidium Iodide/Annexin V Apoptosis Detection Kit APC (Affymetrix eBioscience) according to the manufacturer's instructions. A total of 25,000 events were analyzed for each sample by flow cytometry using the BD Accura C6 equipment and software. For cell cycle analysis, the PI/RNase Staining Buffer (BD Pharmigen) was used according to the manufacturer's instructions. Cell cycle FCS files were additionally analyzed using the Dean‐Jett‐Fox model in the FlowJo software.

### Immunofluorescence staining

A375 cells were plated (800,000 cells per well) on coverslips in six‐well culture plates. After 24 h, the cells were infected as indicated above. After 48 h, coverslips were transferred to 12‐well plates. Cells were washed in PBS and fixed in PBS‐containing 4% paraformaldehyde for 30 min at room temperature. After further washing with PBS (containing 0.1% BSA), cells were permeabilized with blocking PBS‐containing 0.3% Triton X‐100 and 10% Normal Donkey Serum (NDS) for 45 min. Cells were incubated with polyclonal goat anti‐T7 epitope antibody (LSBio, 1:200) in blocking buffer (PBS, 1% BSA, 1% NDS, and 0.3% Triton X‐100) for 1 h at room temperature, followed by incubation with donkey anti‐goat Alexa Fluor 568 (Invitrogen, ThermoFisher Scientific, 1:500) for 1 h. The cells were washed in PBS, stained with DAPI‐Fluoromount‐G (Southern Biotech), mounted on glass slides, and observed under confocal LSM700 microscope (Zeiss). GFP, Alexa 568, and DAPI signals and images were analyzed with the ZEN software (Zeiss).

### Mass cytometry analysis

Prevalidated and pretitrated metal‐conjugated antibodies were purchased from Fluidigm. The metal tag selection was optimized using the Maxpar panel designer (Fluidigm). Cells (grown in 10 cm dishes, see the cell proliferation assay section) were washed three times with PBS, incubated with Versene for 5 min at 37°C, washed again, and transferred to FACS tubes. After centrifugation, cells were resuspended in PBS and counted. In order to eliminate debris and most part of dead cells, samples were submitted to a standard Ficoll extraction protocol, before being resuspended in PBS and counted again. Stainings were conducted according to Maxpar staining protocols. 3.10^6^ cells of each sample were resuspended in serum‐free medium containing Cisplatin‐^194^Pt (Fluidigm) at final concentration of 5 *μ*mol/L and incubated for 5 min at 37°C. Cisplatin staining was quenched by washing the cells with prewarmed PBS supplemented with 10% FBS. After a second wash with PBS, cells were fixed in 500 *μ*L of Maxpar Fix 1 buffer for 10 min at room temperature, centrifuged, and resuspended in 50 *μ*L of Maxpar cell staining buffer (CSB). Samples were mixed with an equal volume of master mix of metal‐conjugated antibodies directed against cell surface markers, that is, ^170^Er‐anti‐EGFR (clone AY13)/^156^Gd‐anti‐CD140b (PDGFRB) (clone18A2) and left for 30 min at room temperature. Master mix was prepared as recommended by the manufacturer (final dilution of antibodies 1:100, 1 *μ*L of pretitrated antibody diluted in 50 *μ*L of CSB for each sample). After one additional washing with CSB, samples were placed on ice for 10 min to chill. Cells were then further permeabilized by adding 1 mL of prechilled 70% methanol. Following 15 min incubation on ice, cells were washed twice with CSB, resuspended in 50 *μ*L of CSB, and mixed with an equal volume of master mix prepared as above containing the following antibodies: ^168^Er‐anti‐Ki67/^167^Er‐anti‐pERK1/2 (T202/Y204) (clone D13.14.4E)/^152^Sm‐anti‐pAKT (pS473) (clone D9E)/^149^Sm‐anti‐p4EBP1 (T37/46) (clone 236B4)/^164^Dy‐anti‐CyclinB1 (clone GNS‐1)/^158^Gd‐anti‐CyclinA (clone BF683)/^143^Nd‐anti‐p53 (clone 7F5)/^159 ^Tb‐anti‐p21/WAF1 (clone 12D1)/^176^Yb‐anti‐cMyc (clone 9E10)/^169^Tm‐anti‐GFP (clone 5F12.4). Following 30 min incubation at room temperature, 500 *μ*L of DNA intercalator solution (125 nmol/L final concentration, Cell‐ID Intercalator‐^191/193^Ir, Fluidigm) was added to each sample. After additional 15 min incubation at room temperature, cells were washed with CSB and twice with deionized water. Pellets were stored at 4°C. Prior to mass cytometry analysis, cells were adjusted to 2.5–5.10^5^/mL in deionized water, mixed with EQ four element calibration beads (Fluidigm) containing ^140/142^Ce, ^151/153^Eu, ^165^Ho, and ^175/176^Lu, and filtered with cell strainer caps. Samples were run on CyTOF 2 mass cytometer (Fluidigm). Normalized data were first analyzed with software available through Cytobank (www.cytobank.org). Live singlets were gated on Cis‐^194^Pt staining (dead cells exclusion) 23 and ^191/193^Ir staining (DNA content, debris, and doublets exclusion). viSNE maps and SPADE spanning trees were generated using 12 markers shown in Figure 3. The tSNE default parameters were as follows: iterations: 1000, perplexity: 30, and theta: 0.5. Data in FCS format were exported to the software environment R for graphic production and statistical computing.

### Statistical analysis

The reported data from mass cytometry analysis were obtained from samples for which the final amount of cells analyzed were within the same range (>20,000 live singlets, see Figure 2A). The *n* value for each subpopulation is indicated above the star plots. Expression distribution for each marker was compared between samples using median values. The significance of differences in distributions was also estimated using a two‐sided *t*‐test (differences in mean values). The *P*‐value was calculated for each paired comparison between GFP (control) and HuR overexpressing cells in excipient‐ and vemurafenib‐treated cells and for each marker.

## Results

### The emergence of paradoxically proliferative subpopulations in low‐dose vemurafenib‐treated A375 melanoma cells is prevented by HuR overexpression

**Figure 1:**
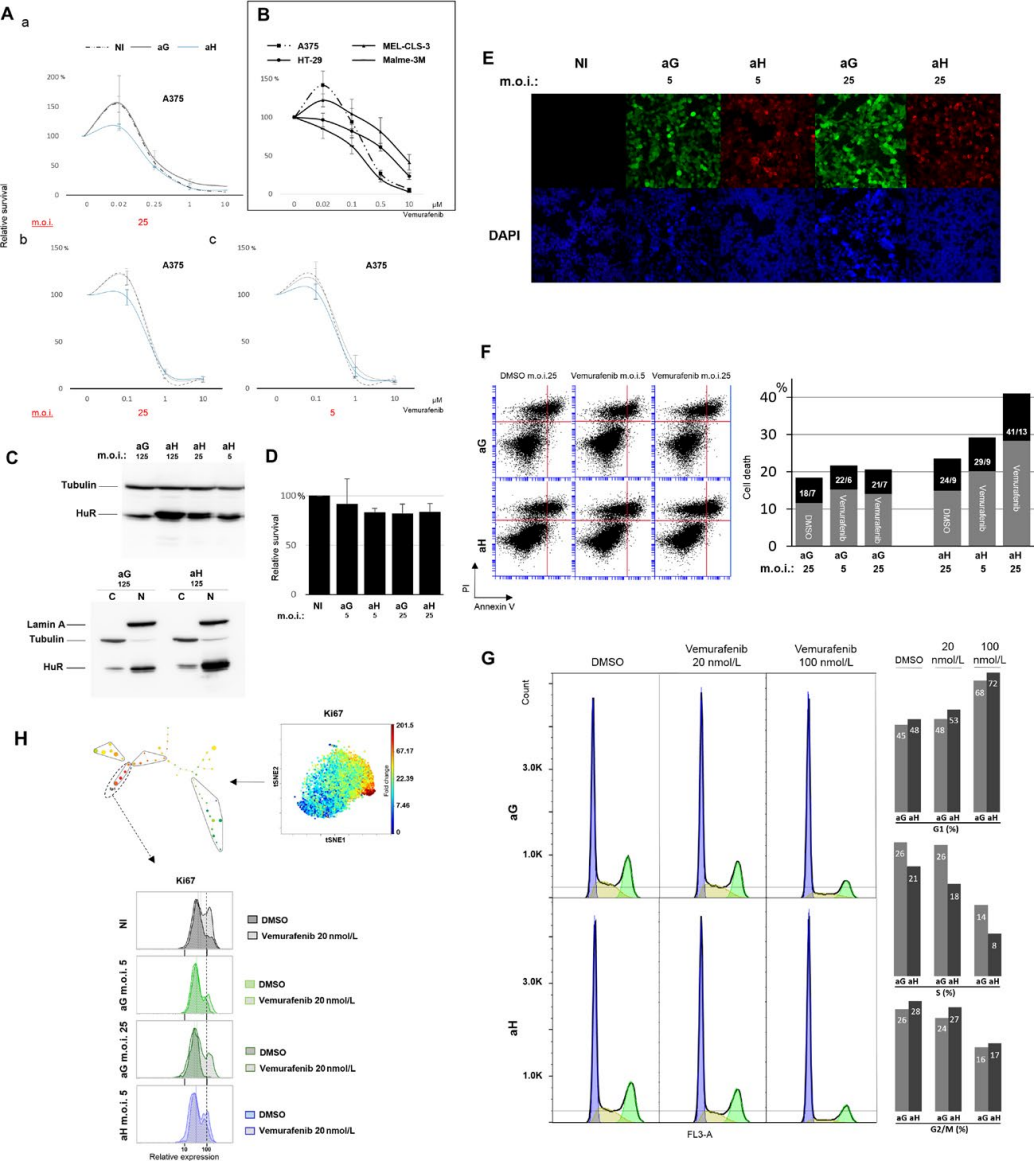
(A) Vemurafenib dose response of A375 cells comparing noninfected cells (NI, dotted line) with the control adenovirus expressing GFP (aG, gray line) or the T7 epitope‐tagged HuR adenovirus (aH, blue line) infected cells: a to b comparison indicates an inverse dose effect of vemurafenib on paradoxical proliferation. b to c comparison (performed in the same experiment) indicates a dose‐suppressive effect of HuR overexpression on paradoxical proliferation. (B) Vemurafenib dose response for various BRAFV600E‐sensitive melanoma cell lines (A375, Malme‐3M) and BRAFV600E‐resistant melanoma (MEL‐CLS‐3) and colon carcinoma (HT‐29) cell lines: paradoxical proliferation is observed at low dose in A375 and to lower extend in MEL‐CLS‐3 cells. (C) Top panel: Western blot analysis using a mouse monoclonal antibody (3A2) on A375 whole‐cell extracts infected with aG or aH at the indicated multiplicity of infection (m.o.i.). Bottom panel: Western blot analysis of HuR expression in A375 cytoplasmic (C) and nuclear (N) compartments following aG or aH infection (shown for the highest m.o.i. used). Note the slight tag‐induced shift in aH samples. An additional second upper band (tagged HuR) is clearly visible especially in cytoplasmic extracts in aH samples. (D) WST‐1 cell proliferation assay: at both indicated m.o.i. values, the proliferation rates of the aG‐ or aH‐infected A375 cells are not significantly affected compared with noninfected (NI) cells. (E) GFP fluorescence in aG‐infected cells and T7 epitope tag (Alexa 568) staining in aH‐infected cells at the indicated m.o.i.: more than 90% of the cells are stained homogenously even at the lower m.o.i. value. Cell nuclei were stained with DAPI (blue). Note that HuR (T7 epitope tag) stains primarily in the nucleus. (F) Cell death effects of HuR overexpression in low‐dose vemurafenib‐treated A375 melanoma cells: flow cytometry analysis of double‐stained (propidium iodide and Annexin V) A375 cells infected with aG or aH at the indicated m.o.i. and treated with either excipient (DMSO) or vemurafenib (100 nmol/L). A total of 25,000 events were analyzed for each sample. Black portion of the histograms represents the percentage of Annexin V‐positive cells within the percentage of dead cells (gray plus black). Their percentages are indicated in the black portion of the histograms. (G) A375 cells infected with aG and aH at m.o.i. 25 and treated with vemurafenib at the indicated concentrations were stained with propidium iodide for cell cycle analysis gated on live cells (blue: G1 phase, yellow: S phase, green: G2/M phase). Corresponding percentage of cells in each phase is indicated with histograms. (H) viSNE and SPADE single‐cell mass cytometry analysis of the Ki67 proliferation marker shown for noninfected (NI) excipient‐treated A375 cells. The color scale line gives an indication of the fold change in expression. For the Sp1 fast‐proliferating cells, the distribution of Ki67 expression is shown for NI cells (gray), aG cells (green, at the indicated m.o.i. values), and aH cells (blue). Hatched curves correspond to vemurafenib‐treated cells (20 nmol/L). Note that, although not detected in the initial proliferation assay (D), a small vector‐induced inhibitory effect is detectable in aG cells (at both m.o.i. values, compared with each other and to the NI cells). This inhibitory effect does not prevent the occurrence of paradoxical proliferation. The aH‐infected cells at m.o.i. 25 are not shown (See Fig. 2 legend). The dashed line helps the comparison.

We have repeatedly observed that some BRAF‐mutated sensitive melanoma cell lines treated with low‐dose suboptimal vemurafenib (i.e., 20 nmol/L and up to 100 nmol/L depending on the cell lines) may paradoxically proliferate. This paradoxical proliferation was reproducibly observed in the A375‐sensitive melanoma cell line (Fig. 1A panel a) as opposed to some other sensitive or resistant BRAF‐mutated cell lines (Fig. 1B). Depending on the experiment, the proliferation rate of A375 cells increased up to 50% at doses of 20 nmol/L vemurafenib, and to a lower extent at 100 nmol/L, at which the expected inhibition also occurred. We then prepared a Cre‐*lox* recombined 22 adenovirus construct for the efficient overexpression of a T7 epitope‐tagged HuR (aH). A similar vector‐expressing GFP was also prepared as a control (aG). aH‐induced HuR expression was checked as being efficient in both nuclear and cytoplasmic compartments (Fig. 1C, bottom panel). To determine the optimal adenovirus multiplicity of infection (m.o.i.) for the overexpression of HuR without affecting the A375 proliferation rate, we first conducted a series of assays to verify that the m.o.i. used in our experiments (Fig. 1D) did not significantly affect the proliferation rate of the aH‐ or aG‐infected A375 cells (aH and aG cells) compared with the noninfected cells. The aH and aG virus preparations were similarly titrated (90% and 100% positive staining, respectively, at m.o.i. 5 and 25 for both constructs) and cells were homogenously stained (i.e., infected) with either construct, including at the lowest adenovirus concentration (m.o.i. 5) used in our experiments (Fig. 1E). As shown in Figure 1A, strikingly, no vemurafenib‐induced paradoxical proliferation was observed in the aH cells in contrast to the aG or noninfected cells. This suppression of paradoxical proliferation varied with the level of HuR overexpression (comparison of panel b with c).

To confirm this effect, we tested for an equivalent difference in terms of the death rate or the cell cycle profile of these cells. As shown in Figure 1F, the death rate was higher in aH cells than in aG cells at 48 h posttreatment (data at 24 h not shown) with a dose‐dependent effect similar to that observed in the proliferation assays, varying with the level of HuR overexpression. Annexin V staining indicated that some dead cells were late apoptotic cells. However, the percentage of Annexin V‐positive cells among the dead cells was not affected by HuR overexpression. Cell cycle analysis using propidium iodide flow cytometric staining was performed at m.o.i. 25, the value at which the maximum difference in death rate occurred (Fig. 1G). The main difference was a lower percentage of cells in the S phase in aH‐ than in aG‐treated cells. This difference depended on the vemurafenib concentration. There was no corresponding cell percentage increase in the G1 phase or decrease in the G2/M phase. Therefore, in aH‐treated cells, G2/M accumulation is likely more important and death rate most likely higher in the S phase. It is important to notice that these moderate percentage differences in death rate and cell cycle profile are consistent with the expected ones from the proliferation assays.

We then conducted single‐cell mass cytometry analyses to identify various subpopulations within the A375 cell line. As an additional confirmation of the observed suppression of paradoxical proliferation, the expression of the proliferation marker Ki67 analyzed in the high Ki67 (fast‐proliferating) subpopulation (Sp1), extracted from one of the conducted experiments, is shown in Figure 1H. In noninfected and aG cells treated with 20 nmol/L of vemurafenib (at m.o.i. 5 called hereafter as aGt and at m.o.i. 25), Ki67 expression was higher than in similarly treated aH cells (at m.o.i. 5 called hereafter as aHt). Noteworthy, a slightly higher Ki67 expression was observed in excipient‐treated (DMSO) aH cells (at m.o.i. 5 called hereafter as aHnt cells) compared with the excipient‐treated aG cells (at m.o.i. 5 called hereafter as aGnt cells).

### HuR overexpression affects subpopulation size within the heterogeneous cell response

**Figure 2:**
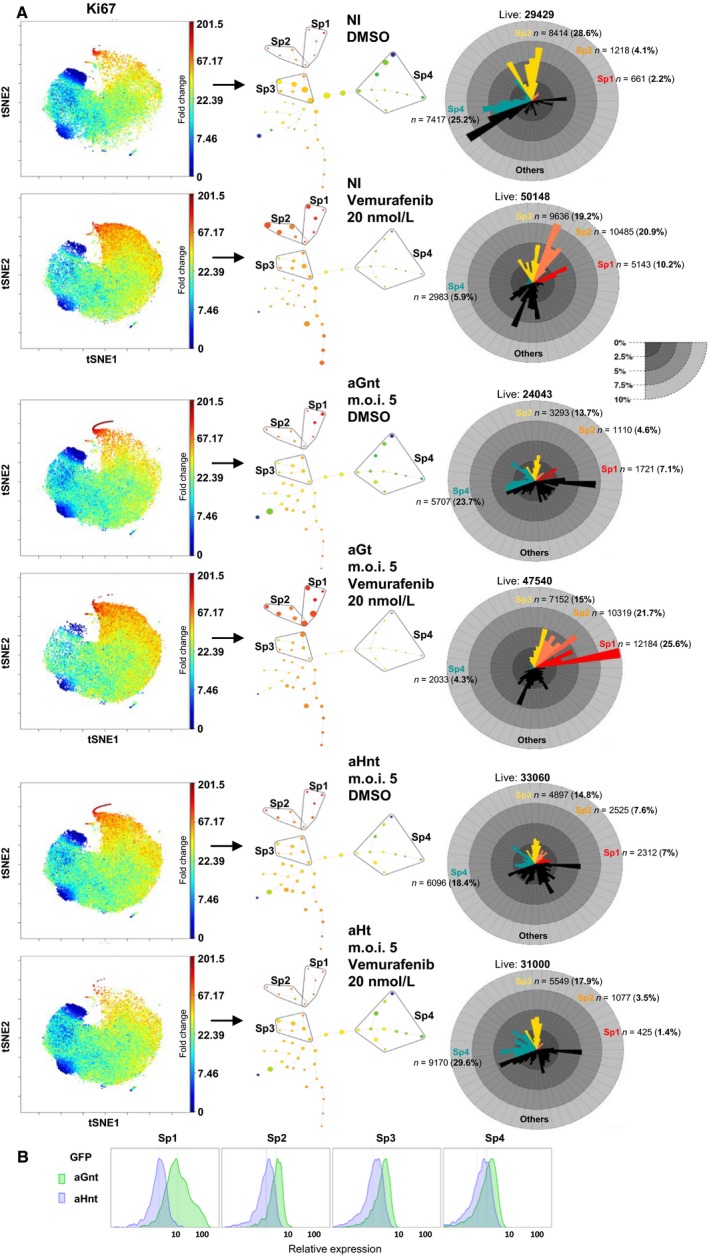
(A) Mass cytometry analysis of the low‐dose vemurafenib‐induced paradoxical proliferation and its suppression in HuR‐overexpressing A375 cells: viSNE maps for NI excipient‐ (DMSO) and vemurafenib‐treated cells, aG (m.o.i. 5) excipient‐ (aGnt) and vemurafenib‐treated (aGt) cells, aH (m.o.i. 5) excipient‐ (aHnt) and vemurafenib‐treated (aHt) cells, with regard to Ki67 expression show an increase in the size of the high Ki67 cells in NI vemurafenib‐treated and aGt but not aHt cells (red/orange dots in the top‐middle area of the maps). The SPADE spanning tree generated from viSNE maps was used to select four subpopulations (Sp1, Sp2, Sp3, and Sp4): the size of the dots is proportional to the number of cells which is also visible in the adjacent star plots. The color of the dots is an indication of the relative Ki67 expression level (based on the median value for each cluster), from red (high) to dark blue (low). Star plots indicate the size and proportion of each subpopulation (Sp1: red, Sp2: orange, Sp3: yellow, Sp4: blue) and each cluster within them for each spanning tree. Note the overlap zone between Sp1 and Sp2 subpopulations, due to the common included cluster. Initially, we intended to compare excipient‐ and vemurafenib‐treated A375 cells infected at both validated m.o.i. values (Fig. 1D). However, repeatedly following the successive centrifugation steps that are needed to conduct the mass cytometry analysis, a large amount of cells were lost, possibly due to cell fragility in the vemurafenib‐treated aH cells infected at m.o.i. 25 (currently under investigation). This precludes any valid further statistical analysis for these samples. The data shown are therefore those obtained from samples for which the final amount of cells analyzed with mass cytometry were nearly similar (the number of live cells in each subpopulation is indicated above each star plot). (B) GFP marker distribution expression in aGnt and aHnt cells (used as the negative control), shown as an indication of the sensitivity of the mass cytometry analysis.

Mammalian cell lines, although often of clonal origin, are composed of heterogeneous cells both in terms of genetic expression 24 and phenotypic behavior, that is, renewal capacity 25. Based on such observations, we tested whether the vemurafenib‐induced paradoxical proliferation of A375 cells arises from cell heterogeneity, prompting an immediate adaptive response of a subset of cells to suboptimal vemurafenib exposure and whether HuR overexpression precludes the occurrence of such adaptive response. To test this hypothesis, a simultaneous analysis of various markers must be conducted at the single cell level 26. Although fluorescence‐based flow cytometry allows the simultaneous analysis of up to 15 parameters, it is limited by the need to compensate for spectral overlap and the background due to cell autofluorescence, and may therefore lack sensitivity to detect small differences within an apparently homogenous cell population. Therefore, we used single‐cell mass cytometry 27 to detect discrete variations in the expression pattern of markers either involved in the mechanism of reversible adaptive resistance to BRAF inhibitors: pERK1/2, pAKT, epidermal growth factor receptor (EGFR), platelet‐derived growth factor receptor‐*β* (PDGFRB), phospho‐eIF4E‐binding protein 1 (p4EBP1) 4, 5, 6, and/or in cell cycle control and functionally identified as HuR targets: cyclin B1, cyclin A, p53, p21, and c‐Myc 15, 28, 30. Ki67 was included as a marker of proliferation, cisPt194 was used to exclude dead cells 23 and also to detect dying live cells. GFP was used as a quantitative sensitivity marker (Fig. 2B). Additionally, p53/p21 and pAKT/p4EBP1 pairs were also used to control for coupled variation within subpopulations (i.e., biologically coherent patterns).

**Figure 3:**
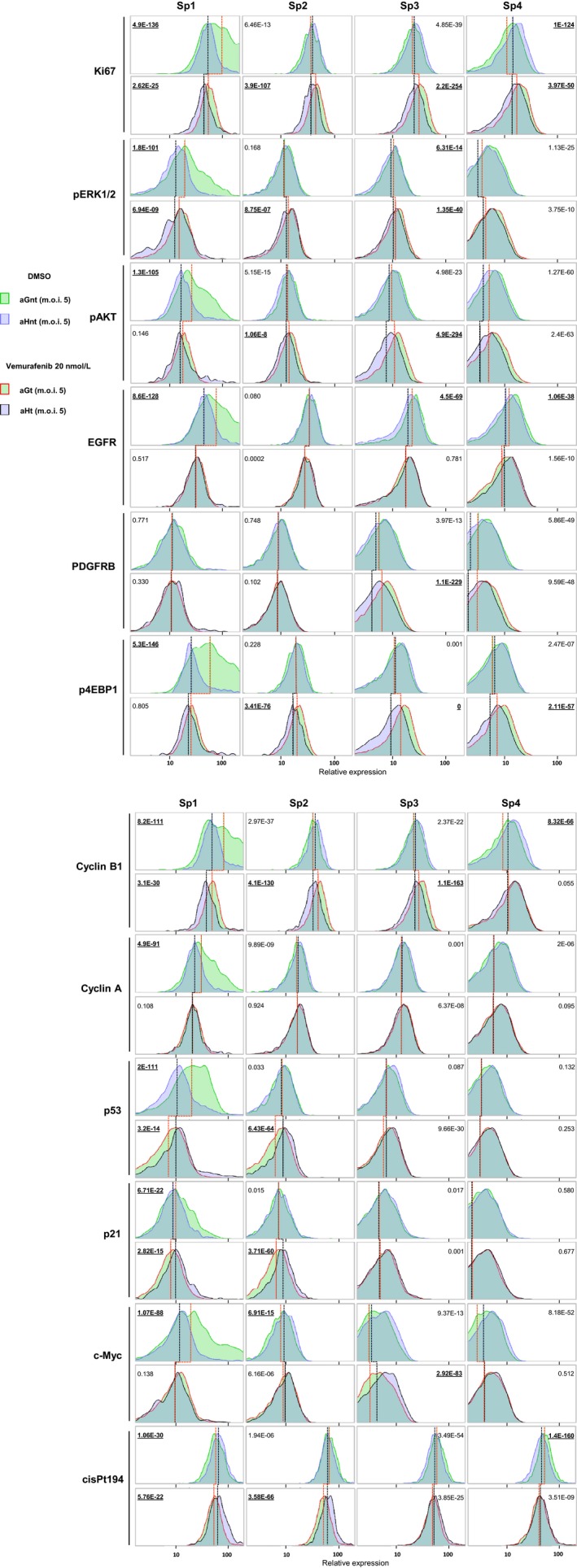
Comparative analysis of each of the four subpopulations, defined in Figure 2A, based on the distribution of the expression levels in the 12 markers used to generate the viSNE maps, shown for: aGnt/aHnt cells (green/blue histograms, top row of each marker, red and black lines for comparisons of median value), aGt/aHt cells (red bordered green/black bordered blue histograms, bottom row of each marker, red and black lines for comparisons of median value), aGnt/aGt (green/red bordered green histograms, comparison between top and bottom row, red line for comparison of median values), and aHnt/aHt (blue/black bordered blue histograms, comparison between top and bottom row, black line for comparison of median values). The *P*‐values (*t*‐test) for paired comparisons are underlined for differences considered significant.

We first ran a viSNE (visual interactive Stochastic Neighbor Embedding) analysis on gated live singlets from samples enumerated below. viSNE maps high‐dimensional data as a scatter plot 31, in which each dot represents a cell and its position reflects the information from all the original dimensions, that is, the 12 markers shown in Figure 3. Figures 2A and 3 describe an experiment different from the one shown in Figure 1H. For the Ki67 marker (Fig. 2A), viSNE maps for the noninfected and aG cells showed a very clear increase in the size of the high Ki67 subpopulation in the vemurafenib‐treated compared with the excipient‐treated cells, contrary to aH cells, in which the size of the high Ki67 subpopulation was even reduced under treatment. We then generated from each viSNE map, a spanning‐tree progression analysis of density‐normalized events (SPADE) which uses a hierarchical clustering algorithm to generate a spanning tree in an unsupervised manner. The target number of clusters to be reached was specified as 50. The Ki67 spanning trees for excipient‐ and vemurafenib‐treated noninfected cells and aGnt, aGt, aHnt, and aHt cells are shown in Figure 2A. Within them, based on the arborescence, four subsets were selected, potentially representing the spectrum of heterogeneity (Sp1, Sp2, Sp3, and Sp4 subpopulations). Based on Ki67 expression levels, the selected subpopulations were recognized to be fast proliferating (Sp1), intermediary mild proliferating (Sp2), slow proliferating (Sp3), or as quiescent/slow proliferating (Sp4, in which some of the clusters were Ki67 negative). In noninfected and aG cells, the size of both Sp1 and Sp2 subpopulations was larger in the presence of vemurafenib than in its absence, contrary to aH cells, in which the size of these two subpopulations even appeared reduced under treatment (star plots, Fig. 2A). Note that the size of the Sp4 quiescent/slow‐proliferating subpopulation was larger in aHt cells than in aHnt cells, but smaller in aGt cells than in aGnt cells. Overall, these data confirm that HuR overexpression can overcome the increase in the proportion of the most rapidly proliferating subpopulations that is induced upon suboptimal BRAF inhibition in the A375 cells.

### Discriminating sensitive from emerging resistant subpopulations

Using the experiment shown in Figure 2A, we conducted a more qualitative and comparative analysis of the four subpopulations defined above, based on the expression profile of the 12 markers used to generate the viSNE maps. Comparative expression distribution and median value comparisons are shown in Figure 3. Lower Ki67 expression in aGt than in aGnt cells in Sp1 subpopulation clearly indicated that this fast‐proliferating subpopulation is sensitive to low‐dose vemurafenib treatment, whereas the Sp2 mild‐ and especially the Sp3 slow‐ and Sp4 quiescent/slow‐proliferating subpopulations paradoxically proliferated under vemurafenib treatment. In contrast in aH cells, no such paradoxical proliferation was seen (this difference between aG and aH cells regarding the effects of vemurafenib treatment in mild/slow‐proliferating subpopulations is hereafter called HuR‐specific pattern). Note that HuR overexpression alone (in excipient‐treated cells) induced a cytostatic effect in Sp1 subpopulation, but had a proliferative effect in the Sp4 quiescent/slow‐proliferating subpopulation. This proliferative effect is equivalent to the one seen in Sp1 subpopulation in the experiment shown in Figure 1H (see above). In Figure 1H experiment, Sp1 subpopulation was defined as fast proliferating, however, the comparison of Ki67 expression range between the two experiments in aGnt cells indicates that the proliferation rate of Sp1 cells in Figure 1H experiment is equivalent to the one observed, in Figure 3 experiment, in Sp2 or Sp3 cells. These differences related to variations in the overall cell proliferation rate between the two experiments explain also the variability seen in the proliferation assays in Figure 1A. The consequence is a difference in the hierarchical clustering and partitioning of cells. As an example, Sp1 subpopulation arborescence, in the spanning tree shown in Figure 2A, is more developed than the one shown in Figure 1H.

pERK1/2 expression was lower as expected in aGt than in aGnt cells, in the Sp1 vemurafenib‐responding subpopulation, but did not differentiate the subpopulations according to the HuR‐specific pattern. Instead, there was an even increased expression of pERK1/2 in aHt (compared with aHnt cells) similar to the one seen in aG cells in the Sp2 and Sp3 subpopulations. As expected, pAKT and p4EBP1 followed each other pattern across the samples (biologically coherent patterns). Importantly, pAKT in Sp3 subpopulation and p4EBP1 in Sp2 and Sp3 subpopulations did follow the HuR‐specific pattern, so did PDGFRB in Sp3 subpopulation. In contrast, EGFR did not follow the HuR‐specific pattern, but in Sp3 and Sp4 subpopulations, aGnt cells showed a higher EGFR expression level than aHnt cells. Regarding markers directly involved in cell cycle regulation and/or functionally characterized as HuR targets: Cyclin B1, could differentiate the Sp2 and Sp3 subpopulations according to the HuR‐specific pattern. This was not the case for Cyclin A which may be due, considering that S phase contains a large fraction of Cyclin A‐expressing cells, to the reduced number of cells in the S phase observed for aHt cells (Fig. 1G). Regarding p53 and p21, their expression pattern appeared as expected synchronized in the Sp1 and Sp2 subpopulations (biologically coherent patterns) in which, in contrast to other markers (Ki67, pERK1/2, Cyclin B1), a right tail of higher expression was clearly maintained in aHt compared with aGt cells. This pattern means that under vemurafenib treatment, the higher HuR expression allows these two targets to be better expressed in the fast‐ and mild‐proliferating cells in which a potentially more efficient cytostatic or cytotoxic effect is expected to occur. Indeed, in addition to the more efficient cytostatic effect of vemurafenib indicated by Ki67 staining in Sp2 subpopulation, cisPt194 staining (Fig. 3, last marker), which indicates the death tendency, was higher in aHt cells in Sp1 and Sp2 subpopulations compared with aGt cells. This observation is consistent with the above‐described size reduction in these two subpopulations in aH‐treated cells. Finally, c‐Myc staining was not discriminative regarding the paradoxical proliferation of any of the subpopulations, but a higher expression level was detected in aHt cells, mainly in Sp3 compared with aHnt cells.

## Discussion

These observations indicate that HuR overexpression can suppress the immediate heterogeneous response to low‐dose BRAF inhibition and the subsequent paradoxical proliferation that occurs in some subpopulations of the sensitive A375 melanoma cell line. In the fast‐proliferating vemurafenib‐sensitive and the mild‐proliferating treated cells, HuR overexpression was associated with increased expression of p53 and p21, increased vemurafenib‐induced death tendency, and a reduction in population size. In the slow‐proliferating cells, HuR‐induced suppression of paradoxical proliferation was associated with the decreased expression of pAKT, p4EBP1, and PDGFRB, but not pERK1/2. These patterns corroborate previous studies showing that the increased expression of pAKT and its downstream target p4EBP1 discriminate sensitive from adaptive/resistant melanoma cell lines, better than pERK1/2 expression levels 4, 6, and are also consistent with the established notion that overexpressed surface receptors like PDGFRB are involved in the mechanisms of adaptive resistance in melanoma 4, 5. We did not observe any increase in EGFR expression in any subpopulation in vemurafenib‐treated cells, however, HuR overexpression was associated with a lower EGFR expression and an increased proliferation rate in the quiescent/slow‐proliferating cells. Consistent with this observation, previous studies have shown that A375 cells engineered to express EGFR proliferate slower 5. Although EGFR expression conferred, in these studies, a selective advantage to A375 vemurafenib‐treated cells, this advantage may occur only upon continued selective conditions and may not be involved in the immediate adaptive response.

It is well known that, at optimal dosage, BRAF inhibitors induce paradoxical proliferation in *BRAF* ^*WT*^ primary melanoma cells 32, and even at suboptimal dosage in some *BRAFV600*‐resistant melanoma cell lines, as it appears in reported experiments 6. To our knowledge, its occurrence in *BRAFV600*‐sensitive cell lines is neither reported nor characterized, as done here, using a single‐cell analysis approach. We have documented the significance of our observation regarding its relation to the early development of an adaptive resistance, by showing that the relative expression profile of the subset of cells that paradoxically proliferate mimics some of the expression traits described among the mechanisms of adaptive resistance to BRAF inhibition. Most studies on the resistance mechanisms and the reversible adaptive response of melanoma cells to BRAF inhibition have been conducted on cell lines artificially homogenized for these resistance traits through chronic vemurafenib exposure. Although this methodology is efficient for obtaining clearly defined differences between sensitive and resistant cell sublines, it does not reflect the emergent and constantly reversible dynamics of heterogeneous adaptive resistance. In a small percentage of cells, adaptive resistance traits most likely exist prior to any selection pressure, or appear during the early stages of the selection process. Therefore, any molecular strategy developed to prevent the occurrence of reversible adaptive resistance, like initiated in our study, must be tested during the emergent phase rather than the established phase of resistance, that is, during the phase when the underlying molecular mechanisms involved in the selection process are at play 26. In vivo, this heterogeneous response and selection process occurs, in an undetermined and possibly variable small proportion of malignant cells, and is difficult to detect in any experimental animal model of grafted or spontaneous melanoma. This is because cell plasticity and adaptive reversible resistance to BRAF inhibitors occur in the environment in which the malignant cells are initially selected for their fitness. Ex vivo, the heterogeneous response to BRAF inhibition in a sensitive cell line can clearly only be tested at low‐dose inhibition as done here. Suboptimal paradoxical proliferation as observed in cell culture might also be pertinent and occur in vivo. Indeed, an insufficient drug penetration and distribution in solid tumors is a yet unrecognized cause of resistance to targeted therapies 33. Interestingly, it has been recently shown that plasma vemurafenib concentration has an impact on tumor response and tolerance in advanced *BRAFV600* melanoma patients 34.

In our single‐cell study experiment, HuR overexpression differentially affected various subpopulations within the same melanoma cell line and this effect depended at least in part on the proliferation rate of these subpopulations. The suppression of the paradoxical proliferation in slower‐proliferating cells may be linked to the proliferative effect of HuR on these subpopulations. Slow‐proliferating cells are often considered to be the pool of cells giving rise to drug resistance. This assumption is based on the available studies conducted in various malignancies 35 and, regarding the resistance to BRAF inhibitors in melanoma, based on the observation that resistant cell lines tend to have a slower doubling times compared with sensitive cell lines 35. Finally and also consistent with our data, the already mentioned study regarding the EGFR‐induced slow‐growth phenotype of *BRAFV600* melanoma cells that may foster the emergence of resistance cells, compared to fast‐proliferating cells, under prolonged BRAF inhibition 5. Our study has therapeutic potential. HuR overexpression or increased nucleocytoplasmic shuttling is drug inducible 37, 38. Many existing drugs could be screened for such effect and then tested in combination with BRAF inhibitors within the experimental setting described here.

## Conflict of Interests

The authors have no conflicts of interest to disclose.
